# The Examination of the Patient for a Homœopathic Prescription*Condensed from report of Bureau of “Homœopathics,” I. H. A., June, 1888.

**Published:** 1888-10

**Authors:** P. P. Wells

**Affiliations:** Brooklyn, N. Y.


### THE EXAMINATION OF THE PATIENT FOR A HOMŒOPATHIC PRESCRIPTION.*

P. P. Wells, M. D., Brooklyn, N. Y.

The first duty of the healer in his clinical office is to ascertain what there is in the present condition of the patient which he is expected to cure; to acquaint himself thoroughly with all the facts of the case, with all their concomitants. This duty is not only first in the order of proceeding, but is second to no other in importance in its relation to the issue of his labor. It is a *sine qua non* in every case of specific (*i. e.*, homœopathic) prescribing. Till this knowledge is obtained no other step can be taken, as all other and subsequent steps are based on this. It is this knowledge of the facts of the case which enables the prescriber to proceed from these to his materia medica and in the facts of this record to find the simillimum to the facts of the sickness. To search here for a curative of any case, without this prerequisite knowledge is to hunt in the dark for a simillimum to an unknown quantity. We speak here, of course, of prescribing under the guidance of the law of *similars*. Prescribing outside of this law, whatever may be arrogated to it by false claims of its "scientific" character, is only, and ever, mere guessing, and always guessing in the dark. There is no therapeutic light available to man except that which comes to him through this law. The arrogance and conceit of old physic only serve to blind the minds of its votaries, and delude them with the conviction that their guessing is, somehow, sanctified and justified by their baseless claim that this blind struggling in the regions of the unknown, to grasp phantoms only existing in their own imaginations, is the embodiment of all there is of the "scientific" in practical medicine. It is certain that guessing is all they have to substitute for a *science* of therapeutics, and as this is so conspicuously poor in itself, and in its results, they seem to be without other resource than loud clamorings, in their claim for their guessing of a "scientific" character! "Scientific"

---

*Condensed from report of Bureau of "Homœopathics," I. H. A., June, 1888.

guessing! Is ignorance or conceit capable of a greater absurdity?

In the two schools of practical medicine of to-day, this first duty is equally recognized as paramount. But there is this radical difference in the views of the two as to their reasons for this. To the one it only suggests a *name* for his case; to the other the points gathered are indices pointing to the curing agent. The one ceases inquiring when he has gathered sufficient facts to justify his *name*, however few these may be. He stops here because he has no further use for facts. With the few he can make his *diagnosis*, *i. e.*, give his name to his case, and this name suggests to the prescriber certain internal conditions, which, in turn, suggest certain drugs as likely to benefit these supposed conditions. These given, and the circle of old-school therapeutic duty is complete. This series of *guesses* it is, which constitutes all there is in that which is so boldly and blatantly proclaimed to the world as the whole of "scientific" medicine! And perhaps the most remarkable fact in this connection is, those who thus clamor the loudest seem to be wholly unconscious of the emptiness of this silly pretense, and appear to be not at all ashamed of it, though there is no "science" in it, but only guessing and a Greek name!

As opposed to this, the other school cannot stop inquiries till *all* the facts are brought out, because if any part be omitted from the record, it may be that in this omission are the facts most important in the *diagnosis of the remedy*. At this point we have the two schools as far apart from each other as possible, in this first and paramount duty. The one investigates for a *name*, the other for a *curative*. The one is content with *few* facts, if these justify his *name;* the other must have *all*, because *all* are necessary to determine the remedy for the case. This necessity is one of the underlying principles of the philosophy of specific medicine, and without this no practical superstructure founded on law can be raised. The elements, and *all* the elements, of the sick condition must be known before any other step can be taken for its cure.

We have said gaining this knowledge is the first duty of the practical healer. We add, it is the most difficult of execution; this difficulty is only equaled by its importance. All in specific healing depends for its successful issue on the faithfulness and thoroughness with which this duty is performed. The difficulty and importance of this duty are so great that no care or labor devoted to it can exceed the demands true philosophy and intelligent conscience make for these in its discharge. These are so great that no margin is found here for haste, carelessness, or indolence. The difficulty is so great that it can only be overcome by endeavors guided and controlled by the most perfect and orderly system of procedure. *Systematic* is the word which should characterize these endeavors from the beginning to the end. This necessitates a plan of procedure at the beginning, which shall insure the survey of the whole field of symptomatic facts before this duty can be accepted as complete, or as a proper basis upon which to search for a curative.

When Hahnemann recognized the relationship of law between sickness and their curatives, as existing in the likeness of the facts of the one to that of the record of the facts of the other, he saw, and was the first to see, the absolute necessity of a knowledge of *all* the facts of the case to be cured, because till this was gained there could be no such comparison of factors as will disclose the likeness which the natural case of cure demands, before any drug can be selected as the specific curative of any case. It is indispensable that this knowledge of *all* the facts of a case shall be in possession of the prescriber, and be made the basis of his therapeutic proceedings before any treatment of his can be brought into the category of homœopathic prescribing. Any attempt at this with partial knowledge of these necessary facts is only sham, and if it be claimed for this that such practice is of the homœopathic school, the claim for this sham is but a false pretense.

The necessity of a systematic plan of procedure in endeavors to compass a knowledge of the facts of sicknesses before seeking for curatives was clearly seen by Hahnemann, and he gave in his *Organon* a sketch of such plan which no subsequent teacher has supplanted or greatly improved. Some, who would be teachers, and have been ambitious of the reputation of models of homœopathic healers, have abandoned this plan and have gone rather for the shorter and easier method of old physic, like this, making names the objectives of their inquiries. They have not been those who by their practical successes have contributed to the evidence which confirms the truths of homœopathic philosophy, nor have they been of the number who have added aught to the power which has extended the triumphs of this through the world. It is rather among this number that a want of partial successes has caused discouragement and doubt, and these in the end have led to apostacy in the few instances where this has disgraced those who sought for homœopathic successes by a neglect of its philosophical principles, and substituting for these, often, a very poor imitation of that easier method which, without law, is content with guessing, while the imitation quiets his conscience, if he has one, and his self-complacency, if he has none, with the boast that "he will use all possible means for the cure of his patients;" not caring to remember, or not knowing, that it is not necessary, in any curable case, to use "all possible means," but only "right means," for the cure, and that these are always and only found in the most similar record of some drug to the phenomena of his sick case. This he presents as evidence of broad "liberality" which knows no prejudice and is confined to no narrow bounds. He would have this accepted as evidence of his superior knowledge of means of healing, whereas it only proclaims his inability before the problem of the discovery of the *right* means for his desired cures. This found, and no others are needed or useful.

The systematic plan of procedure for the discovery of the facts of sickness which, under the guidance of law, discloses the true curative, first observes all which is perceptible to the prescriber in the appearance, manner, and actions of the patient. If in bed—his position; is he quiet or restless; does he change his position often, or does he avoid all motion; his respiration, is it hurried, or normal in frequency; is it in due symmetry with the frequency of the pulse, and in the duration of inspiration and expiration? The expression of the outlook—is it tranquil, excited, or desponding; or what, if any, is the change from that which is natural to the patient? The eyes—are they bright and sparkling or dull; are they injected or clear; are the lids swollen or natural; is the face pale or red, or neither; is it hot or cold, wet, damp or dry? The general surface—is it hot, warm or cold, perspiring or dry; if there be eruption—of what kind, not how has it been named, but how does it look, what are the morbid phenomena attending it? the name is of no consequence to the prescriber in the duty he is now supposed to be engaged in. The voice and spirit—how are these affected, if at all, and what, if any, are the modifications of their normal character? The moral and intellectual functions are to be noted as to all aberrations from their normal state. Has the disposition become, since the sickness, querulous, angry, complaining, easily taking offense, weeping, or sad, as it was not before? The intellect—is it more active or duller than has been its wont; are its perceptions and judgments clear and normal, or are these under the false impressions of delirium? If so, what is the form the aberration assumes? Is the delirium mild or violent, talkative or reticent; is the speech clear and distinct, or is the enunciation imperfect; are answers given promptly or are they delayed and slow, or answers wholly refused; is the imagination vivified by visions which have existence nowhere else; does this delusion talk to, or reach out to imaginary objects in the air? In short, whether in intellect or disposition which is a departure from the natural state of the sick one is a necessary part of the case to be examined into, and is to be, in exactness, a part of the record which makes one side of the equation in every homœopathic prescription, which solves the problem of a cure when it has found in the materia medica record the counterpart of the recorded facts of the sickness to be cured. In this record the aberrations of intellect and disposition are to have a conspicuous place, and careful consideration, before the solution of the specific remedy is decided.

When the perceptible phenomena of the case have been recorded, and not before, then the prescriber will listen to the history of the case, from the patient first, if possible, and then from the friends, if they have additional facts to contribute. Never allow the two to talk at the same time, or either to create confusion by interrupting or correcting the statements of the other. If the case in hand be a chronic disease, it may be necessary to carry the inquiry into the history of the case back into that of the patient's ancestors, in the endeavor to reach a knowledge of the true origin and character of the case under examination. For example—What diseases have been prevalent in the family of the patient? What were the sicknesses which have carried off those who have passed away, if there have been deaths of relatives. This knowledge is often of the utmost importance in discussions of the treatment of such cases. For some diseases are transmitted from parents and grandparents to their children, and the like proclivity to certain forms of sickness are found in individuals of a common ancestry and the clue which leads the true healer to a knowledge of his simillimum is not seldom found in the health history of some progenitor of the patient. In pursuing this inquiry it should be kept in mind that inherited sicknesses often pass the first generation of descent to reveal themselves in the second, or, perhaps, in one even more remote from the original sufferer. The true healer will therefore be very careful and persistent in his inquiries into the origin in the remotest ancestry, if need be, of the chronic case he is to treat.

It may not be necessary to carry the inquiry into the history of uncomplicated acute cases into that of the patient's ancestry. But in cases complicated with the action of aroused chronic miasms it may be of utmost importance to do so. Cases are met sometimes where the clue to their simillimum is only found in this inquiry. This followed up, not unfrequently the cure of the acute attack and of its complicating miasm may be found in the same remedy.

In uncomplicated cases, the inquiry may begin at the first element of the sickness which the patients or friends noticed as the initiatory of the attack. What were the circumstances and conditions in which this appeared and the modalities which accompanied it? And the same inquiries are to be made as to each of the succeeding elements, as also as to the order of time in which they appeared, till a knowledge of all is gained.

The questioning of the history being completed, that of the aberrations of functions in the sick case may begin. And here systematic procedure is indispensable to the required thoroughness and accuracy of the inquiry. This must have a beginning, middle, and end, and all between must be surveyed, that no fault of function may escape detection. Each aberration is to be questioned as to time of appearance, circumstance, and condition attending this, and with the modality which have accompanied its history, with especial reference to all causes, conditions, and circumstances which aggravate or relieve suffering.

With this plan of procedure, where shall we begin? There is no better order for the prosecution of this plan than that of the scheme adopted for the record of the materia medica. This begins at the head and from this follows a natural anatomical arrangement of succession to the end.

The mental and moral symptoms which we have noticed under the division of objective phenomena may perhaps as well, or better, have their place here with the other brain symptoms. After these, the pains or heat, whatever of abnormal sensations may be present in the head, as vertigo, throbbing; noises, as the chirping of insects, etc.; fullness, tension, etc. The pains are to be questioned as to the kind and exact location of each. Then the phenomena of the scalp, if any, are to be noted. Then the organs of the special senses, as of sight, hearing, and smell, both as to function and change in appearance or tissue. The face, as to color, expression, or pains. The mouth and throat, including teeth and tongue, as to appearances which are abnormal, and pains or unnatural sensations, together with whatever modifications there may be of speech, as difficulty or fluency, hoarseness or shrillness, or total loss; of taste, as sweet, bitter, sour, or a total loss, or diminished or exalted state of this function. Then of the digestive function—note all abnormalities as to appetite, thirst, desires, and aversions as to various articles of food and drink; all pains or morbid sensations developed during the process of digestion; all eructations, regurgitations, nausea, and vomitings connected with the food or drink, or which particular articles of these. Then pains or other morbid sensations in the stomach or its associate organs in the process of digestion, not originating in the food or drinks, or in the process of this function. If there be nausea, independent of the digestive process, what is the exact seat of this, the abdomen, epigastrium, or throat? What aggravates or relieves this? If vomiting, what are the substances ejected, and by what is this excited or relieved, and by what concomitants is this attended? What of the hypochondria as to pains or other abnormal sensations, swelling, or sensibility to pressure? What of change in the hepatic organ, if any? The external abdomen, what as to its form? Is it full and round, or flat and sunken? Is it distended? If so, is it by gas, water, or morbid growths, or by retained fecal intestinal contents? If there be pains, what is their exact character and location? How are these affected by circumstance and condition, and what are their concomitants? What of the function of defecation? Is this retarded? If so, what is the character of the evacuated material as to color, form, large or small? Is it dry and hard, or the reverse? What is the character of the impediment: is this in the nature of the material to be expelled, or in a diminished force of the expelling power? What, if any, are the concomitants of the constipation, as hemorrhoids or fissures, or other morbid process in anus or rectum?

Then the urinary organs and functions. Pains in these organs, if any, are to be investigated as to their exact character, location, and concomitants. The secretion, as to quantity, frequency of calls to discharge or the reverse, color, odor, sediment; the sensations on passing the water, their character and location, whether in the vesica or urethra?

The sexual organs and functions are to be questioned as to integrity of tissue and normality of function.

The respiratory organs and air passages are to be investigated as to pains and abnormalities of function. The respiratory act—are inspiration and expiration in symmetrical proportion? Is this performed chiefly by the diaphragm or the intercostal muscles, or by both? Is it accompanied by pain, and if so, what is its character and location? Auscultation and percussion, though of value chiefly to diagnosis and prognosis, are not wholly useless to the therapeutist. For example, in pneumonia, if these disclose the fibrinous exudation of that process already accomplished, certain remedies are excluded from the treatment, being no longer curative of this inflammation after it has passed this process, no matter what other symptoms there may be. If there be a cough—what is its character? Is it dry or loose, with or without expectoration? If with, what is its character, and is it raised with ease or difficulty? Is the cough seldom or frequent or constant? Is it short and slight or violent and in protracted paroxysms? What are its conditions and concomitants?

The exterior conformation of the chest—are the two sides in symmetrical development? Are the sub-clavicular spaces rounded out or hollow? Are the intercostal spaces distended or normal? Are there pains—if so, what is the character and location of them? Are they increased or unaffected by respiratory or other motions?

The spinal column is to be questioned as to deviations from normal structure, as to pains, if there be any, as to their exact character and location, and conditions of aggravation and relief. If there be any other abnormal sensations, as sense of heat or cold, these are to be carefully noted.

The extremities are to be questioned as to whatever of pains or embarrassments or loss of motion.

The skin—as to eruptions or modifications of its transpiratory function, temperature, etc.

Sleep—sleepiness, sleeplessness, with causes and concomitants. Dreams, as to their character.

Febrile phenomena, as to time of accession, and concomitants. The symmetry of the elements of the paroxysm, or the predominance or absence of either.

The general phenomena, as to temperament, disposition to take cold, or to be especially affected by any particular cause of sickness, or habits of body which predisposes to any particular forms of sickness, as for example, rheumatism, neuralgia, or spasms of any kind, causes which aggravate or relieve general pains or sufferings. The special character of general pains, as pressing, burning, boring, drawing, shooting, fixed, etc. The period of exacerbation. Acuteness, dullness, or loss of general sensation, or whatever change there may be in this function. How are the general phenomena affected by change of air, *i. e.*, in the open air or in a room? How by motion or repose? How by eating, drinking, sleeping, or by the performance of any bodily function? Whatever of sick phenomena which are dependent for existence on the change of function of no particular organ. And these general phenomena are not to be overlooked, overshadowed, obscured, or their importance underestimated, because some particular local suffering, or derangement of some particular function has chiefly had the attention of the patient or his friends, and has by them been regarded as the one object of the prescriber's attention. To relieve the suffering of patients is, of course, one objective of the physician's endeavors, but as indicia pointing to the means which most certainly and speedily relieve these, the greatest pains are not always the most important. On the contrary, these are not unfrequently found in the general symptoms, where they are so easily overlooked.

Having by this process of examination of functions and general phenomena gathered the requisite "totality of the symptoms" of a case, how shall we proceed, through them, to find our specific curative? There are two methods practiced by doctors who equally claim to be recognized as practitioners of specific medicine. One is to infer from the gathered facts a certain general condition of the patient or of certain of his organs or of their functions, and having some regard to the law of similars, infer that a certain drug or drugs produce similar conditions, and therefore this, or these are the similar agent or agents the law requires for the cure. This *inferred* or *imagined* condition of the patient the doctor calls the *pathology* of his case, and having proceeded thus far on the basis of the totality (and having, probably, been satisfied with less of examination and fewer symptoms than a strict compliance with the demand of the law required), he is fully satisfied he has fulfilled the duties of a *specific* and "scientific" practice. The two are here so beautifully harmonized and brought into such perfect fellowship, he is more than satisfied, he is delighted. The *truth* is, in all this, the prescriber has not been loyal to either. He has given in this proceeding so much to guessing as demonstrates his lack of loyalty to *specific* medicine; and has shown so much, though but a partial, regard for the law of the similars, as to thoroughly disgust the myrmidons of old physic, from whom, by this clumsy imitation of their method, we seem to have been seeking "recognition." This is the *wrong* method.

The *right* differs from this in that it takes this whole group of facts, clean and naked, stripped of all theory and inference, and goes to the materia medica record for the most similar group, in the recorded proving of some *one* drug. This found and given, as the law demands, and all has been done for the case which specific medicine requires, and all which is needed for the cure of any curable case. This is what it is to practice, in a word, with the homœopathic law, which practice with aught added to or subtracted from, this is not. Such practice, before the simplicity and truth of pure Homœopathy, is no better than the thrice beaten straw which is cast out to be trodden under foot by the most stupid and filthy of animals.

---

DISCUSSION.

Dr. Gee—This is a very able paper, and one of practical importance to all of us. As the Doctor said in the beginning, an article of a similar character, that is on the same subject, is found in Dunham's *Therapeutics*, presenting, perhaps, a scheme somewhat after the same plan, but not going into the details as fully and deeply as Dr. Wells has done; and certainly a paper of this sort on our desks in the shape of a slip or card would be a very great help to all of us in the examination of the patient, as we are apt to overlook some things perhaps in the order of questioning. One thing in Dr. Wells' paper occurred to me which perhaps needs a little explanation, and that was his allusion to the excellent remedies in pneumonia. I don't know that we can draw a line that is distinctly marked and say that after the exudation has taken place, any remedy or set of remedies may not be indicated. I can see that in a nervous disturbance calling for a remedy just as the key of a Yale lock is necessary and the only thing that will open the lock—that remedy may be called upon. I can hardly see that we can exclude a remedy without some explanation. Perhaps the Doctor did not make it as clear as he could.

Dr. Wells—That point was put down understandingly, and to illustrate I would state that after the deposit of fibrine has taken place, Aconite is never indicated, and never has been since the world was made; it is of no use and never will be. Meeting the points suggested by my friend Dr. Gee, of nervous symptoms calling for a remedy; those are best met by remedies which are related to the symptoms, and the *condition* of this stage of pneumonia, which is revealed by auscultation and percussion. These revelations *are symptoms* which it is not wise to overlook or neglect. The remedies which are so powerful and beneficent in the first stage, *i. e.*, before that of the deposit into the lung tissue of the inflammatory product are now no longer in place, and if their use be longer persisted in, the result will often be pernicious and always attended by loss of time. It may be we sometimes restrict the meaning of the word

*symptom* or symptoms to too narrow limits. *Objective* symptoms are *facts* equally with *subjective*, in which class are found the revelations of auscultation and percussion, which, in best treatment of pneumonia, are never neglected.

Dr. Gee—It seems to me the Doctor has misunderstood ; that borders very closely upon pathological prescribing. We do know that patients suffering from pneumonia have a nervous anxiety, an anxiety that will be indicated by the expression of the face; the heart will show weakness. In such a condition Aconite might be indicated, and if so, I would certainly give it.

Dr. Wells—I think the only difference between my friend Dr. Gee and myself is that I am attempting to take in the whole view of the case and he seems to take in only a part, and that seems to me the difference. Aconite seeming to be indicated has led me into that blunder a hundred times, and a hundred times has led me to disappointment. But to find a remedy necessary for these new accessory symptoms you must secure a remedy for them, and it is best to secure it at one stroke, than to go around the corner for it.

Dr. Kent—Take a case of pneumonia that has advanced to the stage of exudation and let that patient get a little cold sufficient to arouse him to a state of mental anxiety. With a superficial examination you will find Aconite indicated, but just as sure as you give it you will fail. Give Sulphur at once and you will cure your patient. Never mind the fact that Aconite has the superficial show, I say in ninety-nine cases out of one hundred give Sulphur When I first commenced to prescribe I gave Aconite and I never had anything but failure, and have been fooled many times by giving it.

Dr. Allen—Do you prescribe Sulphur in the second stage, or rather in the exudative stage, when the patient has taken a little cold and become nervous under those symptoms ?

Dr. Kent—No, sir.

Dr. Nash—If the Aconite symptoms are present, are we taught anywhere to ignore them and base our prescriptions on the fact that the disease has passed to the second stage ? I think not. I think, with Dr. Wells, that we will very seldom find indications for Aconite there, and that we should look further. It may be Sulphur, as Sulphur is a remedy used in the exudative stage. So we must ignore Aconite and look further, and we will find our failure arose from a very superficial examination that seemed to indicate Aconite. It is a fact that many patients come into our offices who can be prescribed for at once. A patient may have eaten too much ice-cream, and we know at once the cause of their trouble, and we know in a short examination what they require, and we can prescribe for them very quickly. When we have chronic cases and we find the case cropping out again after we thought we had cured it, then it is absolutely necessary that we must go through this very process that Dr. Wells has been describing. One of the best rules I have found is one that Hahnemann gave for examining a patient, and that is: Never ask the patient a question that can be answered by yes or no, because if we ask such questions as, "Have you a pain in the head?" especially of a nervous person, they always have it, and you could ask them if they had a pain almost anywhere and they would always have it. But if we call on them to tell their symptoms in their own language, and not allow them to answer yes or no, we find it a very great help.

Dr. Holmes—This paper of Dr. Wells' has been one I wanted to hear. I am a new member of this Society, and I have come a long distance to attend this meeting, with the hope I might learn things that would be of great advantage to me. Now, the examination of the patient is a subject that I must confess I am a little lame on. The great trouble is how are you going to find time to do this? Supposing a man has afternoon office hours of only two hours' duration, or an evening hour of one hour's duration, and in that time crowds in twelve, fifteen, or twenty patients, and perhaps out of that number there are two, three, or five patients that need just such an examination? To me it has been impossible to do it. I am not, like Lippe, a flash prescriber. Another thing, we do not get paid so well where I come from for that work. Say we get fifty cents for that work [laughter], and it takes the whole time to examine the patient, and we cannot give the time and attention to other patients that are waiting.

The President—I think in reference to the examination of a patient, that as the younger members of the profession become more and more acquainted with the manner of meeting a patient their time spent in an examination will be shorter than when they began. I believe, too, that the examinations and the value of them will improve as they grow older and more experienced in listening and in asking. I know that my own experience has been very similar to yours; that the time required for first examinations seemed excessively long; but the older I grow and the more chronic cases I have to deal with the more positive I am that the time spent in the first examination is the best spent time. Hahnemann tells us that when the first examination is well made two-thirds of the work is done. It is unfortunate that any one who represents so fine and beautiful a profession as Homœopathy should be placed under conditions where such poor compensation is received as most of us do receive and particularly the younger members of the profession. To many it is a matter of bread and butter, of course, but there is a higher motive than bread and butter, and that is to learn how to do the thing correctly; improve yourself, and get yourselves into positions where you can become so proficient as to command proper compensation for your work. My advice to the young men is not to slight your chronic cases, but rather say to your patients, if you are pushed for time come to me on Sunday morning and I will give you an hour or more, even if you pay me nothing for it, and if I find the remedy and I cure you that will be compensation enough for me. What I was going to say in regard to the quickness and rapidity of decision or insight of some prescribers or some men who have grown with their work is this. I once saw my father, who was in a very great hurry to go somewhere, when a young, thin, and lank man came to him and said: "Doctor, I want to see you." My father replied, "I have no time now; you must come again." "But I want you to attend to my eyes right away; I have some very sore eyes here, and I want them attended to." "How long have you had them?" "Only a week." "Let me look at them a moment," and with a rapidity that was perfectly marvelous, my father told him what was the matter with him. The young man had symptoms and my father showered them on him, and they suited so well that this man stood in consternation and said: "Who told you all about me?" That was artistic. He cut that thing short. Of course, it was the wrong way to do it; but it was the way he did it at that time. He did not ask that man have you got this and that, but he said you *have* got that; you are worse in the morning; you are cross, irascible—a cross fellow—and he said I will give you a powder, and he gave him Nux vomica. Of course, I do not recommend that to you; I only wish to cite that instance of a person who knows a remedy, that is, materia medica, and how he can get quickly at a set of questions without making direct questions that will cut short an examination very much; and, as Hahnemann once said—he never printed it—show me the examination of a sick person and I will tell you if the man knows anything about materia medica. This little story I have just told I don't want to be anything more than only an instance of the knowledge of a remedy and going right at it in a very quick way, in a way I do not wish or advise any one to imitate or prescribe on,

I only wish to show how quickly you may take such a patient and go through his Nux vomica symptoms. All these things that you learn by careful examinations are not lost time.

Dr. Biegler—I would like to add a word or two to the foundation Dr. Wells has given us for our work. The manner in which Dr. Wells has given us this outline for our work is invaluable to all, but especially to the young man. If they, the young men, will take this as a foundation for their work, I will guarantee that it will not be very long before their fees will be largely increased, and that almost voluntarily. The first prescription made right, based upon the foundation of work, and he can afterward sleep and remain quiet, with his conscience and his mind quiet.

Dr. Wells—I want to say to this young man [referring to Dr. Holmes] and every young man: get no more work than you can do well. Do it well and take time enough for it. There is always time to do work well.

Dr. Allen—I think that Dr. Holmes has struck a very vital point. It is certainly a puzzling point to a young man. My attention has been very forcibly called to this very point within the past month by a consultation I had with a professor of materia medica in one of our homœopathic colleges. A couple of years ago I endeavored to show him by a long and entertaining correspondence, based upon a case already reported in a medical journal, that there was a better way than the way he was doing, and he appeared to be a willing and apt student to learn this better way. A month since I met him at a State Society, and, after a conversation, he says, "I have faithfully tried Hahnemann's method of taking the case, and it won't do for me. It takes too long; it takes up too much time; it don't pay. It may do for somebody else, but it don't do for me." Now, that question that Dr. Holmes puts is right to the point, and we must get over it somehow and in some way, and I do not know of any better way than that which Dr. Wells suggests, that there is always time to do work well, and if Dr. Holmes will turn over a new leaf, and instead of taking ten patients an hour will cut it down to one or two and charge them properly, he will have more to do and make more money, and learn his materia medica faster.

Dr. Wells—I wish to say in the interests of humanity, of all we love most, that when we come to a sick man, a sick woman, or a sick child, we should confine ourselves to the fact and be conscious of it, that we are engaged in so great a duty that money has no place there. [Applause.]

Dr. Stow—It does pay to be careful and as accurate as it is possible for a human being to be in the examination and in prescribing for the sick. There is this thing in it, if nothing more, if at first you expend much time and get small pay and perhaps lose some because you take so much time, depend upon it that the experience you get in looking carefully into the case will make you so expert that you can take care of twice or thrice the number of cases in a very short space of time, and the public will find it out, too. That is one great fact that should be instilled in the minds of all that have doubts in adhering to Hahnemann's rules. I believe it, because I have seen it verified in my own practice time and time again, and I am satisfied that the little success I have had in the practice of Homœopathy has been due to the very fact that where an important case comes in that I do not see into at first sight, I take my pen and paper and record everything about the case—every symptom.

Dr. Ballard—I have had some little experience which goes to justify a man for the time he may spend in the first examination. The question which Dr. Holmes puts is a vital one, and it has not been answered satisfactorily to the side of the bread that has the butter on. But I have worked that way and I have worked the other way, and I find that the best way is the shortest way after all. I had a case of neuralgia in a lady situated on the left side of the face. I made an examination for the case as it presented itself (and this case will sustain Dr. Wells in his Aconite business). I went over these same symptoms—all skin deep; I worked over it for two months. Her friends were all the time advising her to take Morphine; I told her that if she resorted to Morphine that she would likely become a chronic sufferer; that it would not cure. I went to see her one evening and sat down with her and said: "There is something about your case I have not found out yet, and I want to find it out." She could not tell me anything. I went into her history—her private history. She was a widow, a very estimable lady. I asked her about her husband, and I learned that he had been a sailor and a sea-faring man. I asked her if she had ever had eruptions of any kind, and she answered nothing of the kind; never had anything of the kind. I then went from the scalp clear to her feet, over and over, and I examined her finger nails, the hands, the palms of the hands, and I saw three little spots there, and I said how long have they been there, and she said, I don't know; but I have had them a good many years; she then said that skin seems to get dead and peel off. I gave her a dose of Thuja. She had a paroxysm in a little while, the worst she had had. Suffice it to say these paroxysms became less and she would suffer at longer intervals, so that within a week she was all right. She then complained of a sore throat, and on examination I found on the inside of each tonsil as pretty a picture of a chancre as a man ever saw. There was two months wasted in trying to cure that neuralgia because I did not spend the proper time in the first place. Another case: I was called in to see a child. The child lay in its cradle. The skin was hot and dry but not harsh. The child did not want to be spoken to. It had no wants for anything only to be rocked; the cradle must be kept in constant motion. The child would once in a while rise up in this way (describing). I gave Cina, expecting in twenty-four hours the case would be well. The symptom remained. I gave Cina higher; the symptoms continued. The child must be kept in violent motion, *i. e.*, rocking. But these same Cina symptoms became more prominent all the time. The trouble was in the first prescription, I did not properly take my case. I prescribed what seemed to be indicated superficially. My Cina having failed me I go back and find I have a child fourteen or sixteen months old that never walked; it was a fair, plump child. How has the child's health been? I went back to the very beginning, learned the peculiarities of the child in every way, shape, and manner; that if the child could get an egg the child would eat it. A little while—some months before that, it had had blisters on the body. Those blisters had coalesced and all broke out in a big ulcer. I said: "How have the child's head and ears been?" "Oh! soon after that it had a discharge from both ears." "Did the doctors cure that?" "Oh! yes; I injected Carbolic acid and that cured him." Now we have the case. The tubercular meningitis which is presenting itself, is but the suppression of the disease. I had to go back and give Calcarea, which restored the discharge from the child's ears, and the brain symptoms were relieved and the child was cured. These cases simply illustrate what Dr. Wells says, that you may have a case in which at first sight Aconite is indicated. But stop! You find there is something that says, "don't give Aconite;" there is something else needed. Look deeper and you will find it.

Dr. Hawley—I have one suggestion to make to young men. You want to learn in the first place your patient is not going to die in a minute, and if you can't study your case through to-day give him some Sac. lac., take his fifty cents, and have him come again.

Dr. Wells—To any young man in the room I would say, I

knew a young man once who began as others do to try and practice Homœopathy; he did not know anything about it; he had only the *Organon* and materia medica to rely on. I have known that young man to study cases a fortnight, and then he would cure them. Take the time, stick to it, and then you will cure.

Dr. Allen—I began with intermittent fever in that way. It pays to do it infinitely better than it does to make a catch prescription that is more likely to miss than to hit. A short time since a gentleman wrote me from Central Michigan a long picture of his case. I asked for more particulars, and another long letter came, and I was still not satisfied, and I again asked for more particulars. The characteristic symptoms were these: When walking in the house, on the street, on his farm or anywhere, suddenly as though struck by a hammer or by a club, would be a blow on the right side of his head, that would always throw him to the left. The remedy I sent him did not cure him. I then wrote to him to come down and see me (he lived some two hundred miles from me), which he did, and I spent one whole day with him to the neglect of my other patients; but I got what I thought was the picture of his case. I finally found it in Tabacum, and two doses of the two hundredth has made a very different man of him.

Dr. Wells—Next to the importance of taking the case and the selection of the right remedy comes the right use of it. Now I told some one I was not coming here again, but if I do, I will bring a paper on the right use of the remedy. I want to say now, that I have not half learned that lesson. It has been the most difficult lesson of my life. You remember a year ago, when we were down at Long Branch, we went into the discussion of the treatment of suppressed gonorrhœa. I had a young man come to me about three months ago and he came in with a cane, limping, and he could just step and that was all. He had pains in his feet and ankles and he could not walk, and he had been under what was considered homœopathic treatment for two years. He had taken Bryonia and Rhus, and was no better. I had a suspicion about the young man, so I asked him a plain question, and he said "yes." I gave him three months ago one dose of Thuja$^{200}$, and I have given him nothing since but sugar of milk and he is cured. He got that one dose and no more, and the secret was in letting that dose alone.

The President—Was there any reappearance of the original symptoms in that case?

Dr. Wells—There was a return of moderate urethral discharge.

Dr. Ballard—You may remember at Long Branch last year, that Dr. Gee called on me for a case I had under treatment, and I said it was improving under a single dose of Psorinum. That man has never had but that one dose and he is a well man.

