# Errata

**Published:** 1888-11

**Authors:** 


ERRATA.—In last issue, page 511, line 16, for *case* read *law*; page 512, line 12, for *inability* read *imbecility*; page 512, line 12 from bottom, for *spirit* read *speech*; page 513, line 4, for *whether* read *whatever*; page 513, line 12, for *solution* read *selection*; page 514, line 19, for *modality* read *modalities*; page 514, line 15 from bottom, insert *or* after heat; line 8 from bottom, for *or* read *of*; page 515, line 4, for *which* read *with*; page 517, line 9 from bottom, for *we seem* read *he seems*; page 518, first line, omit first *with*.

